# Towards a data publishing framework for primary biodiversity data: challenges and potentials for the biodiversity informatics community

**DOI:** 10.1186/1471-2105-10-S14-S2

**Published:** 2009-11-10

**Authors:** Vishwas S Chavan, Peter Ingwersen

**Affiliations:** 1 Global Biodiversity Information Facility Secretariat, Universitetsparken 15, DK 2100, Copenhagen, Denmark; 2 Department of Information Studies, Royal School of Library and Information Sciences, Birketinget 6, DK 2300, Copenhagen, Denmark

## Abstract

**Background:**

Currently primary scientific data, especially that dealing with biodiversity, is neither easily discoverable nor accessible. Amongst several impediments, one is a lack of professional recognition of scientific data publishing efforts. A possible solution is establishment of a '***Data Publishing Framework' ***which would encourage and recognise investments and efforts by institutions and individuals towards management, and publishing of primary scientific data potentially on a par with recognitions received for scholarly publications.

**Discussion:**

This paper reviews the state-of-the-art of primary biodiversity data publishing, and conceptualises a '*Data Publishing Framework' *that would help incentivise efforts and investments by institutions and individuals in facilitating free and open access to biodiversity data. It further postulates the institutionalisation of a '*Data Usage Index (DUI)*', that would attribute due recognition to multiple players in the data collection/creation, management and publishing cycle.

**Conclusion:**

We believe that institutionalisation of such a 'Data Publishing Framework' that offers socio-cultural, legal, technical, economic and policy environment conducive for data publishing will facilitate expedited discovery and mobilisation of an exponential increase in quantity of 'fit-for-use' primary biodiversity data, much of which is currently invisible.

## Background

Open access to primary biodiversity data is essential both to enable effective decision-making and to empower those concerned with the conservation of biodiversity and the natural world [1]. However, much of the existing primary biodiversity data is neither accessible nor discoverable. The majority of the legacy primary biodiversity data within the mega-biodiversity world is not even digitised [2]. For instance, of the 190 million primary biodiversity data records accessible through the data portal [3] of the Global Biodiversity Information Facility(GBIF), merely 8.1% data is about species distribution of 17 Like-Minded Megadiverse Countries (LMMC) [4]. Over 64% of these LMMC data records are hosted by other countries and international networks [5]. Such a lack of up-to-date, easy, fast, reliable and affordable discovery and access to a wide spectrum of primary biodiversity data leads to an unnecessary duplication of efforts. Verification of results becomes difficult, and investment in research data creation/collection remains underutilised as these data are currently trapped invisibly in institutional and individual cupboards, computers and disks. Furthermore, such a lack of access to primary scientific data is an obstacle to interdisciplinary and international research [6].

Thus, an urgent need exists for the discovery and mobilisation of primary biodiversity data from the developed, developing and under-developed countries, into the public domain. To address this issue, we propose a conceptual framework of 'Data Publishing Framework' together with its phased implementation plan. We put forth the compelling argument that, if implemented, such a framework could bring in much required cultural and attitude changes towards the management and publishing of primary biodiversity data.

### Open access to primary scientific data: the state-of-the-art

Calls for open access to data have been growing since the United States policy statements on data management for global change research or Bromley Principles in 1991 [7]. There are now a number of statements, policies, and guidelines on open access to primary scientific data [8-29]. The Berlin Declaration of 2003 on access to knowledge in the sciences and humanities that calls to promote the Internet as a functional instrument for a global scientific knowledge base has been signed by 266 scientific bodies worldwide [24]. In 2004, the Organisation for Economic Co-operation and Development (OECD) also recognised the importance of open access to primary scientific knowledge [29].

Several national and multilateral agencies, mostly from developed nations have commissioned programmes which aim to facilitate open access to primary scientific data. For instance, Canada has 'Data Canada', the dedicated national infrastructure that has been proposed to assume overall leadership in the development and execution of a strategic plan to encourage access to scientific data [30]. The Chinese Ministry of Science and Technology launched its 'scientific data sharing programme' (SDSP), which has the participation of 24 government agencies. It aspires to facilitate access to 80% of Chinese scientific data to its public by 2020 [31]. The Global Earth Observation System of Systems (GEOSS) 10 Year Implementation Plan [32] explicitly acknowledges the importance of data sharing in achieving the GEOSS vision and anticipated social benefits. It calls for the full, free and open exchange of data, metadata and products.

The majority of the scholarly publishers (e.g. Nature, Science etc.) have joined in implementing the common principle that scientists must make their data available for independent use, without restrictions, once it has been used in publications [33-40]. In the early days of the open access, movement sequence data provided a compelling early example of the power of journal editors to promote the public deposit of scientific data, and of research funders to ensure that biomedical research information is available in public databases [41]. However, these efforts are still to yield any significant results as already existing data remain unpublished and under-utilised [42]. The majority of the initiatives to make scientific data accessible is mostly discipline specific, and has focused on 'big science' rather than 'smaller science' [43]. We do not yet have a good model for coordinating the large proportion of small-scale data originator(s) who produce a huge quantity of primary biodiversity data and thus form the 'long-tail' of science data [44,45].

Further, one should not underestimate the power of data mining for hypothesis generation and discovery of novel hitherto unknown conceptual associations and taxonomic insights in such rich environments as biodiversity data collections. In medical and biological scientific full text databases there have been strong indications of the advantages of applying specific text mining techniques for new drug discoveries, originated by Swanson [46,47] and e.g. further developed in [48].

### Publishing primary scientific data: current impediments

Primary scientific data are the lifeblood of science [49]. However, a host of factors act as an impediment to facilitate seamless discovery and access to primary scientific data. The lack of an infrastructural-technical, socio-cultural, policy-political, legal, and economic 'Data Publishing Framework' that would encourage scientists to publish primary scientific data is often cited as the major constraint. Some of the impediments to open access to research data include (a) concerns about inadvertent misuse of data, (b) lack of ownership agreements, (c) competition for academic position and funding, (d) usability, (e) lack of informed consent and confidentiality [50]. Data are essential both to enable effective decision-making and to empower those concerned with the conservation of biodiversity and the natural world.

While we believe that technical and infrastructural bottlenecks can be easily overcome, more serious impediments consist of socio-cultural and policy-political aspects of the existing 'data publishing mechanism'. The problem at present is a lack of incentive for data originator(s) and manager(s) to go through the work necessary to prepare their data for publication [51]. This ghost of 'what is in it for me?' syndrome seems to be the root cause, which prevents scientists and research institutions to make concerted efforts in the management, archiving, and publishing of primary scientific data.

### Open access and primary biodiversity data: challenges

At the lower end of the requirement spectrum, the discovery of primary biodiversity data sets is essential. This should be applicable, even if the data are sometimes proprietary, require licensing agreements to be signed prior to access, are confidential, demand security clearance, are under temporary embargo until the authors execute their rights of first publication, or for other reasons [52].

With the establishment of the GBIF in 2001, an attempt is being made to develop a global infrastructure to facilitate the discovery, inventory and access to the world's primary biodiversity data. GBIF's mission is to facilitate free and open access to the world's biodiversity data to anyone, anytime, anywhere. Currently, GBIF facilitates access to over 190 million data records through its data portal, . However, these primary biodiversity data records are just a minuscule component of the estimated voluminous amounts of data out there.

For example, over 6500 natural history collections globally are believed to house in the range of 3 billion specimens[53,54], which many experts believe is an under-estimate. Most of the time such calculation is based on the random sampling of major museums in the northern hemisphere, leaving out medium and small sized institutional and individual collections in developed and under-developed regions of the world. Furthermore, a large quantity of primary biodiversity data is collected by a long tail of biodiversity researchers and amateurs [45] that neither have the encouragement nor the infrastructural support (technical, economic, as well as human resources) to manage and disseminate the data generated as a result of trillions of dollars worth of investment. Whilst infrastructure support is increasingly being made available, the lack of a professional recognition mechanism for institutions and individual investment in data management and dissemination still remains. This is becoming one of the major barriers to free and open access to biodiversity data.

## The data publishing framework

### Why a data publishing framework?

The foregoing discussion emphasises the need for a 'Data Publishing Framework' to evolve metrics and indicators that would provide due incentives to multiple actors involved in the data creation/collection to its publication. Both data originators and information systems/networks have emphasised a need for data usage metrics and indicators to ensure that the overall utility and impact of their data management and publishing activities can be objectively documented, leading to their recognition as a scientific activity on a par with the recognition one receives for the actual scholarly publications.

Such metrics should capture the quantitative and qualitative impacts of data management and publishing efforts. The collection, analysis and interpretation of impact metrics and indicators should form an integral part of the data management and publishing cycle.

Without a system of recognition and reward to the collectors, managers, and publishersof primary biodiversity data, we shall continue to rely on the good will or spare time (sic) of researchers to mobilise data into the public domain. Furthermore, measures of scientists' productivity would benefit through data publishing, which requires a cultural change in the recognition of scientific output [55]. Such an incentive mechanism would achieve increased data mobilisation and increased accreditation, both desirable to scientists.

The Data Publishing Framework is not only essential for increased and expedited discovery and mobilisation of primary biodiversity data. In long run it could narrow the gap of uneven distribution of biodiversity data worldwide [54] as data originator(s) irrespective of developed, developing and under-developed part of the globe would be equally encouraged to publish biodiversity data.

### The data publishing framework: components

**Figure 1:**
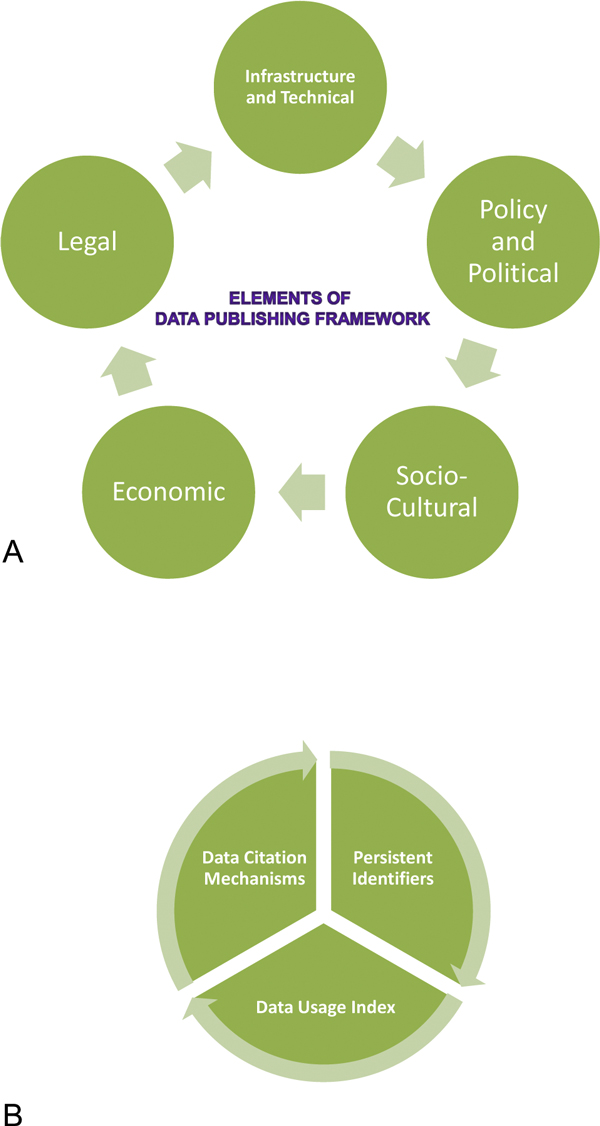
**a - Five inter-dependent and complementary elements of the 'Data Publishing Framework'**. b - Three core technical-infrastructural components of the 'Data Publishing Framework'.

Five elements that constitutes 'Data Publishing Framework includes (A) socio-cultural, (B) technical-infrastructural, (C) policy-political, and (D) legal environments and (E) economic investments for supporting various activities of data publishing cycle (Figure. 1a). Three major technical-infrastructural components, without which meaningful implementation shall remain incomplete are: (i) Persistent Identifiers to data publishers, datasets, the data record itself, as well as to data versioning, and data citation; (ii) a Data Usage Index (DUI) at every access point; and (iii) an effective Data Citation mechanism (Figure 1b).

These elements, as well core technical-infrastructural components are not only complementary, but they are also inter-dependent on each other. For instance, efficient implementation of a 'Data Usage Index (DUI)' requires that data publishers, datasets, data records, including their versioning, be assigned and resolved through 'Persistent Identifiers. Similarly, a Data Citation mechanism requires that each instance of data use and its citation be assigned and resolved through Persistent Identifiers. Thus, these three components must be treated as integral and inseparable aspects of the 'Data Publishing Framework'. No clear sequence of components exists, thus they need to be implemented concurrently. A certain degree of flexibility can be employed with regards to their sequence of implementation.

In the subsequent sections we discuss the possible choice and implementation approach of three technical-infrastructural components of the 'Data Publishing Framework' in the context of discovery and mobilisation of primary biodiversity data. As a hypothetical scenario we have considered the implementation of this framework for data discovered and mobilised through the GBIF as the preferred mechanism. This is simply because the existing GBIF network provides a complex, dynamic, yet functional platform of distributed and decentralised data discovery and mobilisation. However, it would not be restricted to GBIF alone. Such a framework could be implemented within any other domain-specific network irrespective of its size and magnitude of operations. Therefore, implementation of such a framework as described in subsequent sections should not be construed as 'GBIF-o-centric'. In fact, it could be implemented for any free and open, inclusive, community driven, primary scientific data discovery and access infrastructure of local to global scale.

### Persistent identifiers

Persistent or unique global identifier is a short name or character string guaranteed to be unique [52]. It permanently identifies a data set independent of location. The persistent identification of digital resources can play a vital role in enabling their accessibility and re-usability over time [56]. Thus, they form the first and the foremost essential component of the proposed 'Data Publishing Framework'.

Several kinds of persistent or unique global identifiers such as Handles, Digital Object Identifiers (DoIs), Archival Resource Keys (ARKs), Persistent Uniform Resource Locators (PURLs), Uniform Resource Names (URNs), and Life Science Identifiers (LSIDs), etc., are in use [57]. There is a lack of agreement on which is optimal. Furthermore, progress in defining the nature and functional requirements for identifier systems is hindered by a lack of agreement on what identifiers should actually do. Commitment to deploy and reuse globally unique shared identifiers and the implementation of services that link those identifiers is the key to rich integration of distributed datasets [58]. For instance, LSIDs [59] were developed to provide globally unique identifiers for objects in biological databases [60]. LSIDs are the recommended persistent identifiers by the Biodiversity Information Standards (TDWG) [61]. However, uptake to date of LSIDs have been limited with only Universal Biological Indexer and Organiser (uBio) [62], Catalogue of Life [63], the International Plant Names Index (IPNI) [64], and Index Fungorum [65] implementing it.

For the biodiversity informatics community the attractions of LSIDs include the distributed nature of the identifier, the low cost, and the convention that resolving a LSID returns metadata in Resource Description Framework (RDF) model [66]. The latter facilitates integrating information from multiple sources using tools being developed for the Semantic Web [67], although the mechanism for resolving LSIDs is not supported by existing Semantic Web tools. By using the existing DNS infrastructure, LSIDs avoid the need to set up a new central naming authority [67].

In the context of the proposed 'Data Publishing Framework', unique global identifiers should be assigned not only to datasets, but also to its publishers, every individual datum and its author(s), data versioning, and data citation. Further, simplified mechanisms are needed that make it easy for individuals to assign these identifiers to their data [55]. Given the options available, the choice of choosing the suitable unique global identifier should reside with data publishers.

Institutions must recognise that the application and maintenance of unique global identifiers form just one part of an overall digital preservation and publishing strategy. Without adequate institutional commitment and clearly defined roles and responsibilities, unique global identifiers cannot offer any guarantee of persistence, locability, or actionability in the long or short term [58,67].

### Why a Data Usage Index (DUI)?

The DUI is intended to demonstrate to data publishers that their biodiversity efforts creating primary datasets do have *impact *by being accessed and viewed or downloaded by fellow scientists. Dataset providers and publishers, such as the individual scientists struggling to generate and structure single or sequences of records into high-quality primary biodiversity datasets and their host institutions require incentives to continue their efforts and recognition of their usage. In a scientific digital library and open access environment, such as that developed for bibliographic information in Astronomy, usage is measured in a two-dimensional way. The straightforward way is to apply common bibliometric indicators with respect to citation patterns and impact. However, this track is not yet feasible in the case of biodiversity datasets. There exist no standards for dataset citations in scientific papers and quantitative analyses of citations to biodiversity datasets will provide unreliable results. A second avenue is to define usage metrics, based on requests, viewing and downloading of research publications in the form of metadata, abstracts or full text via the digital library client logs [68].

Thus, the proposed DUI for biodiversity datasets is initially intended to apply the second avenue based on GBIF log data, as pointed out above. Because neither a standard data citation or persistent identifier mechanism exists for biodiversity datasets the isolation of actual references to datasets in the scientific literature is extremely difficult at present. Hence, traditional citation analyses are not yet feasible. However, a Data Usage Index consisting of a range of usage indicators extracted from the GBIF and other biodiversity dataset portal usage logs is definitively within reach. The proposed DUI is thus intended to make the (GBIF) dataset usage visible, providing deserved recognition of their creators and to encourage the biodiversity dataset publishers, providers and users to:

• Increase the volume of high quality data discovery, mobilisation and dataset digitisation;

• Further use biodiversity data and information in scientific work;

• Improve formal citation behaviour regarding datasets in research; and

• Develop standardisation of dataset information

The implementation of the proposed DUI is intended to be carried out according to a number of phases, as outlined below, starting with the data extraction from the GBIF main Web portal usage, web services and data dump logs covering 2008.

### What is the DUI?

The proposed DUI consists of usage indicators concerned with

• Unique Visits;

• Loyal Visits (repeated visits by same IP address);

• Viewing of dataset records

• Download of datasets & dataset records

• Volume and (rank) distributions of datasets & dataset records

Since the biodiversity datasets are stored, located and used via the Web a combination of common bibliometric/scientometric issues [69] and webometric analyses [70,71] can be addressed. In terms of the former issues *rank distributions *can be performed on produced datasets and dataset records over providers (institutions, regions, countries) or themes (species, taxa, geo-locations of habitat, etc.). Such distributions are similar to scientific publication analyses, and deal primarily with the volume of the datasets or number of dataset records generated over a specific time period. Clearly, time series of such distributions are feasible and may uncover patterns of dataset generation behaviour.

Most dataset usage indicators are associated with scientometric and webometric analyses, except that so far linking behaviour has not developed with respect to biodiversity dataset use. On the other hand, similar to the Web, dynamics like versioning and additions to already stored datasets are feasible. Usage indicators commonly measure interests, recognition or impact of the objects analysed, via visits, viewing and downloading activities (Nielsen Media, Ratings). By visiting (searching or retrieval) and viewing dataset records one may assume *interest *in the dataset, whilst the volume of downloading volume may demonstrate *usage*. Logging and analysing these activities are common to Web search engine log analyses and the issues of isolation of search sessions done by the same 'user' or 'visitor' [72] (. In the DUI case we initially deal with 'visits' defined by IP address and search activity patterns over specific time windows - not individual visitors.

**Figure 2:**
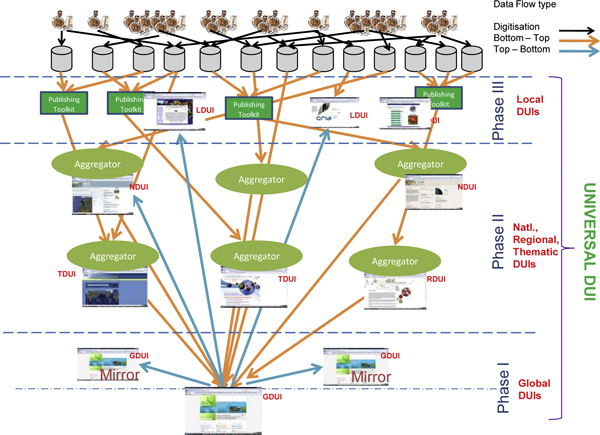
**Phased implementation of the 'Data Usage Index' from global to local data discovery and access points**. Abbreviations Used: GUDI: Global Data Usage Index, RDUI: Regional Data Usage Index, TDUI: Thematic Data Usage Index, NDUI: National Data Usage Index, LDUI: Local Data Usage Index

The DUI indicators may be in the form of *absolute *measures, metrics *normalised *according to stored volume of records, *relative *to something, e.g. the world average of dataset usage measured by average download volume of dataset records across all datasets or selected thematic datasets, or *weighted *according to specific dataset profiles of institutions or countries to be compared. The latter (weighted) indicators lead to dataset Usage Crown Indicators, in line with Crown Indicators for scientific publications and citations [73]. Table 1 demonstrates a number of basic absolute measures and a few selected normalised ones. At a later phase, after a Citation Mechanism and a Persistent Identifier have been designed and implemented, common scientific publication citation analysis and impact metrics can be devised to complement the usage indicator as part of a Universal DUI (Figure 2). A range of comprehensive usage indicators is currently under definition and development by authors and will be discussed in forthcoming articles.

**Table 1:** (Non) - normalised Data Usage Index (DUI) indicators

	**Measure**	**Description**
1	Visits	Number different visits (by IP address)
2	Unique Visits	Number of different first-time visits (by IP)
3.	Loyal Visits	Number of visits repeatedly visiting a unit
4	Download Events	Number of different visits making a download
5	Download Frequency	Number of downloaded records from a unit (also distribution over units) - by visitor type
6	Download Volume	Size in MB - can be averaged
7	Download Impact	Download Freq. *D(u) *over stored records *r(u) *in unit: *D(u)/r(u)*
8	Avg. Download Freq.	Download Freq. per Event *d(u): D(u)/d (u)*
9	Usage Ratio	Ratio of Download Events over Visits *v(u) for unit u*: *d(u)/v(u) *- by visitor type

### The DUI: implementation

Currently available 'primary biodiversity data' has multiple access points. Considering the trend of data publishing activities and the involvement of multiple actors in this arena, it is safe to state that even the upcoming datasets would have multiple access points. This makes implementation of the DUI complex yet challenging. As depicted in Figure 2, data not only flow from contributors to local, regional, thematic, national and global access points, but it also flows equally in a reverse and lateral directions. For instance, data published through the GBIF global data portal is often also accessible through thematic or regional access points, which are contributed by 'data publishers' other than the one who operates/maintains the access point.

We propose a three-phase implementation of the DUI (Figure 2). In the first phase a Global Data Usage Index (GDUI) is published by computing 'data usage logs' at global access points such as the 'GBIF Global Data Portal' at  and its mirrors. In addition to GUDI, a second phase would compute Regional DUIs (RDUIs), Thematic DUIs (TDUIs) and National DUIs (NDUIs) using data usage logs of national, regional and/or thematic access points. The third phase would include computation of DUIs at all levels (GDUI, RDUI, TDUI and NDUI) using data usage logs of all access points. Normalisation of all DUIs (GDUI, RDUI, TDUI, and NDUI) and Local DUIs (LDUIs) would result into a Universal DUI (UDUI), which would be used as a normalised index to compute the 'Data Usage Index' of each participating publisher.

We propose that the DUI be computed on an annual basis beginning with GDUI during the first year, followed by RDUIs, TDUIs, and NDUIs during the second year and the inclusion of LDUIs during the third year leading to a Universal DUI. The implementation of such a multi-level DUIs can be a complex operation. Obvious questions that arise are whether such an exercise happens in a centralised or decentralised manner. We suggest that web services or RESTful services [74] be implemented to harvest the 'data usage logs' of participating publishers by a coordinating agency. Coordination of such an exercise by a coordinating agency would provide much needed neutrality, credibility and acceptability of the DUI by all involved in the data management and publishing life cycle ranging from donors and, publishers to users. At the same time the Citation mechanism and the persistent identifiers for biodiversity datasets are planned to be devised and launched. This implementation should assure the start of common dataset citation impact analyses in a global manner.

### The DUI: improving relevance

**Figure 3:**
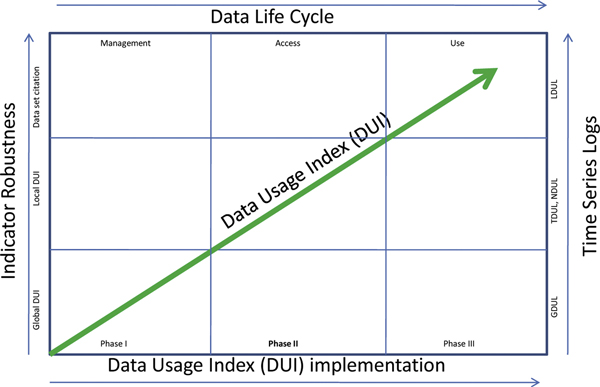
**Essential mechanisms to improve 'relevance' of the 'Data Usage Index'**. Abbreviations Used: GDUL: Global Data Usage Logs, TDUL: Thematic Data Usage Logs, NDUL: National Data Usage Logs, LDUL: Local Data Usage Logs, DUI: Data Usage Index.

Several factors would influence the relevance and acceptance of the proposed DUI. Four major factors that would determine robustness and relevance of the DUI are (i) the implementation phases of the DUI; (ii) temporal richness of data usage logs; (iii) indicator robustness, and (iv) improved data management and publishing cycle. Both relevance and robustness of the DUI would be directly proportional to the implementation of the DUI, temporal richness of data usage logs, indicator robustness, data citation practices and improved DataLife Cycle management (Figure 3).

For instance, as management and access to data improves, data usage would increase both in its diversity and numbers. This would result in more hits to multiple access points of the same data, which in turn would result in an increased number of downloads, citations both in scholarly publications and e-publishing. Similarly, as one implementation phase advances to the next, the number of publishers participating in the DUI exercise would increase. This means that the normalised index would become more and more stable, credible and representative. The same assumption is applicable to a temporal increase spanning multiple years of data usage logs.

### Data citation mechanism

Without an effective data Citation Mechanism the implementation of the 'Data Publishing Framework' would remain incomplete. Thus, universal standards for citing datasets are essential. As mentioned above, currently we lack consistency in data citations, which is sure to provide much needed high visibility to data. It is difficult or impossible given the existing citation metrics system to identify who originally created or added value to a datum [55].

For data to be citeable it is necessary that they can be referred to in a consistent way [51]. Thus, a data citation standard/mechanism should retain the advantages of print citations, be distinguishable from them, add other components made possible by (and needed due to), the digital form and systematic nature of data sets, and should be consistent with most approaches. Further, citation formats need to ensure clear credit/acknowledgement to the originator(s) and linking ability to data sets.

Mechanisms or standards for 'deep citation' or references to subsets of data sets are essential to appropriately acknowledge the creator/collector of data records, which constitute the data set(s) used. Data citation standards need to be flexible enough to accommodate deep citations, versioning, as well as any amount of additional information of interest to archivers, producers, distributors, publishers, or others without losing functionality [52]. An issue around citations of versions of same data sets is critical and needs to be resolved in such a way that links between prior and new versions are functional and consistent. Such a citation scheme should enable forward referencing from the data set to subsequent citations or versions, and even a direct search for all citations to any data set.

Altman and King [52] proposed a standard for citing quantitative data, which has six components, i.e. Author(s), date of dataset publishing, data set title, persistent identifier, universal numeric fingerprint, and a bridge service. One might add that a standard also must include an identifier at the start of reference entry that denotes that the entry is concerned with a dataset, not a scholarly publication or other information type. This goes beyond the technologies available for printed matter and responds to issues of confidentiality, verification, authentication, access, technology changes, existing subfield-specific practices, and possible future extensions. With such a citation standard various components can be permuted to suit different journal styles without loss of functionality.

Though the standards for citing quantitative data proposed by Altman and King [52] address most of the existing challenges, further review and understanding of other options needs to be evaluated. Enriched metadata for datasets is essential for deriving appropriate citations either for the entire or part of the data set. The persistence of the connection between data citation and the actual data depend on some form of institutional commitment.

## Discussion

**Figure 4:**
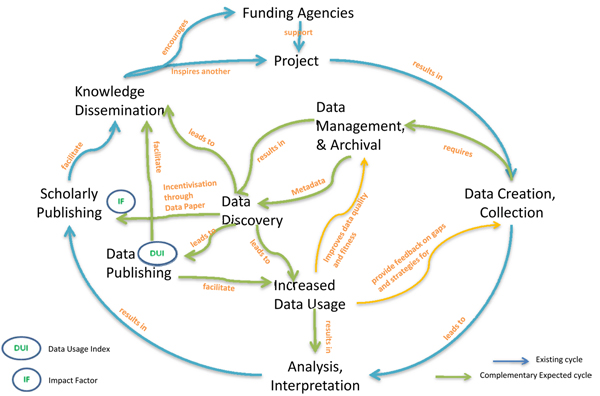
**Anticipated impact of the Data Publishing Framework implementation on scholarly publishing and primary scientific data publishing cycles**.

An early implementation of three basic components of the 'data publishing framework' viz., Persistent Identifiers, DUI, and Data Citation mechanisms would impact the present data and information cycle. However, more importantly it would provide much called for recognition for individual efforts in management and publishing of primary scientific data, in our case primary biodiversity data. In addition to traditional 'Impact Factor' efforts of data management and publishing managers ranging from originator(s), data manager(s), aggregator(s) and publishers at all levels would be recognised through the DUI (Figure 4).

If implemented the proposed 'data publishing framework' would open the flood gates to an increased volume of primary biodiversity data, justifying public investment in biodiversity science and conservation of biotic resources. We believe that a DUI will bring a the long tail of invisible primary biodiversity data into the public domain as scientists and data originators efforts would be recognised on a par with scholarly publications. As shown in Figure 4, datasets could also then be applied for multiple uses other than the single intended cause of creation. This would result in improving 'fitness-for-use' of data, as users are expected to offer the feedback on both optimum quantity and quality of the data. We also believe that both the traditional 'Impact Factor' indicators for scholarly publication and the 'Data Usage Index' for data publishing would act complementarily to each other, the improving their relevance, credibility and robustness synergistically.

For attaining the envisaged outcomes through implementation of a 'Data Publishing Framework' the following priority actions are needed;

(a) roles and responsibilities of researchers (originators), institutions, funders and users must be clearly defined and articulated,

(b) data should be created, collected and managed in accordance with established guidelines and best practices,

(c) models and mechanisms of data management and publishing of data must be both efficient and cost-effective, and

(d) assurance of long term preservation of data [75].

In addition to achieve the rank of an article publication, a data publication needs to meet the two main criteria of consistence and quality [51].

## Conclusion

The implementation of a 'Data Publishing Framework' would, we believe, expedite the process of the archival and curation of an increased volume of primary biodiversity data, as scientists and originators of data would realise the value and recognition of doing so. We believe that the decentralised implementation of 'Data Publishing Framework' on a local-thematic-global scale would indeed build a foundation towards a 'global research infrastructure' for an open access regime in biodiversity and conservation science. We further believe that it would progress the building of a data and information aided 'virtual research space' for future studies in biodiversity [76]. However, to implement the 'Data Publishing Framework' there is a need to bring about a cultural and attitude change on the part of scientific publishers, scientific societies, authors, institutions and funding agencies towards its proactive uptake.

## Competing interests

The authors declare that they have no competing interests.

## Authors' contributions

VC conceived ‘Data Publishing Framework’ and drafted the manuscript. PI developed algorithm for ‘Data Usage Index’. Both VC and PI read and approved the final manuscript.
